# Eccentric Training to Restore Persistent Functional Loss Following Ruptured Achilles Tendon: The Role of Fascicle Length and Muscle Excursion

**DOI:** 10.1155/tsm2/9782280

**Published:** 2024-12-30

**Authors:** Rikke Hoeffner, Rene B. Svensson, S. Peter Magnusson

**Affiliations:** ^1^Department of Orthopaedic Surgery, Institute of Sports Medicine Copenhagen, Copenhagen University Hospital Bispebjerg-Frederiksberg, Copenhagen, Denmark; ^2^Department of Clinical Medicine, Center for Healthy Aging, University of Copenhagen, Denmark; ^3^Department of Physical and Occupational Therapy, Copenhagen University Hospital Bispebjerg-Frederiksberg, Copenhagen, Denmark

**Keywords:** achilles tendon rupture, clinical function, eccentric training, fascicle length, fat fraction, tendon length

## Abstract

**Background:** Persisting deficits are often seen years after an Achilles tendon rupture despite dedicated rehabilitation efforts. A possible reason for reduced function is elongation of the tendon and accompanying shortening of the muscle. Strength training with focus on the eccentric component of loading leads to longer muscle fascicles in healthy persons.

**Purpose:** To investigate if focused eccentric training would result in increased muscle fascicle length, strength and excursion, reduced fat fraction, and functional improvements.

**Study Design:** Longitudinal within-subject exploratory study.

**Methods:** Fourteen persons, with a prior unilateral Achilles tendon rupture who still experienced functional deficits > 1 year after injury, underwent an isokinetic eccentric training program for 12 weeks. Tendon length, muscle fascicle length, cross-sectional area, strength, and functional parameters were measured on the injured and uninjured sides before and after 12 weeks of training.

**Results:** For gastrocnemius fascicle length and fat content in the triceps surae, no significant change over time on the injured or uninjured side was detected. There was a significant interaction effect (*p*=0.0065) and side effect (*p* < 0.0001) for heel-rise count, resulting in a significantly smaller difference between the sides at 12 weeks compared to baseline. With extended knee, the eccentric and concentric plantar flexion peak torques showed a significant interaction effect (eccentric: *p*=0.0074 and concentric: *p*=0.0187), as well as a significant side effect (eccentric: *p*=0.0002 and concentric: *p* < 0.0001) and time effect (eccentric *p*=0.0179 and concentric *p*=0.0093). Between weeks 0 and 12, ATRS significantly improved (mean difference 11.6 points, 95% CI [4–19], *p* < 0.0001).

**Conclusion:** Fascicle length was not altered by the intervention. However, plantar flexion toque, heel-rise count, and, importantly, the patient-reported outcome measure ATRS improved.

## 1. Background

The recovery time after an Achilles tendon rupture is typically 9–12 months, and irrespective of surgical or nonsurgical treatment, a complete functional recovery is often not achieved [1–5], which remains a puzzle. Muscle weakness and reduced function can persist even a decade after injury despite intense and protracted rehabilitation [6, 7]. The tendon may be elongated after a rupture [7], and it has also been suggested that muscle length may impact muscle excursion during a heel-rise [8], albeit scarcely investigated. Recent data show that persons with a functional deficit more than 2 years after an Achilles tendon rupture display an elongated soleus and gastrocnemius tendon, reduced (30%–60%) performance of the heel-rise test, and significantly shorter fascicle length (18%) of the medial gastrocnemius muscle (GM) [9].

Eccentric exercise is beneficial for improving strength [10], and therefore strength training with a focus on eccentrics is popular. Studies have shown that strength training with a focus on the eccentric component leads to longer muscle fascicle lengths [11–13], which may be due to longer resting sarcomere lengths or addition of sarcomeres in series. These studies have examined the effect (on muscle strength, mass, fascicle length, and pennation angle) on hamstring and quadriceps muscles, while effects of eccentric training on the triceps surae muscles are conflicting with either increases in fascicle length and muscle thickness of all three muscles (the medial and lateral GM and the soleus muscle) of the triceps surae [14–16] or unchanged muscle architecture [17].

Most studies have been carried out on healthy individuals, and none have investigated if eccentric training would also result in increased muscle excursion and functional improvements in persons who have had an Achilles tendon rupture. It remains unknown if a muscle that has undergone shortening secondary to elongation of the tendon, and therefore has a reduced overall excursion, can increase in fascicle length and function following eccentric training. We hypothesized that eccentric training would increase fascicle length in persons with Achilles tendon rupture who have been left with an elongated tendon and shortened GM fascicles.

## 2. Methods

### 2.1. Design

This was a longitudinal, within-subject, exploratory investigation of persons with a prior unilateral Achilles tendon rupture more than 1 year after the injury, treated with or without surgery, who still experienced a significant functional deficit. The study was designed to investigate the morphological adaptations of the medial GM and the influence on function. The injured side was compared to the uninjured side before (week 0) and after 12 weeks of eccentric plantar flexion training. Ethical approval was obtained from the regional Ethics Committee (No. H-17039510). All participants were given oral and written information about the study and gave written informed consent. Assessments were performed before and after 12 weeks of intervention.

### 2.2. Participants

Fourteen participants (12 men and 2 women, mean (SD) age: 48 years (8.9), height: 178 cm (5.6), BMI: 27 (4.9)) were recruited via social media posts. Inclusion criteria: an Achilles tendon rupture more than 1 year ago and should answer “yes” to the following two questions: (1) Do you experience that your Achilles tendon injury still hinders your overall daily activities, including recreational activities?, and (2) are you unable to perform a heel-rise of equal height when standing on one leg at a time? Exclusion criteria: Less than 3 cm difference in heel-rise height between the injured and uninjured leg, a history of bilateral Achilles tendon injuries, other injuries or pathological complications in the lower extremities, or any serious health-related problems.

### 2.3. Ultrasound (US)

Fascicle length, pennation angle, and thickness of the medial GM were measured with B-mode ultrasonography (HI VISION Ascendus, Hitachi Medical Systems) using a 100 mm long probe. The participant was lying prone with the knees straight and feet hanging freely over the edge of the examination table [9], and the procedures from a previously described protocol were applied [18, 19]. The probe was aligned perpendicular to the skin and parallel to the tibia and the following images were obtained: (1) medial, (2) central, and (3) lateral at the mid muscle belly. All measurements and quantitative analyses were completed in Fiji/ImageJ2 (Version 2.3.0) using a previously published plugin (simple muscle architecture analysis, SMA) [20] to minimize assessor influence. Two modifications were made to the plugin: (1) The data import method was changed to support our file format. (2) The automated detection of aponeuroses was replaced with manual outlining due to difficulty establishing automated parameters that worked consistently across all recordings [21]. The assessor was blinded to injured/uninjured side.

### 2.4. Magnetic Resonance Imaging (MRI)

The participants were MRI scanned in a Philips Ingenia Ambition 1.5T scanner, software version 5.6.1.2 (Eindhoven, Netherlands). Scans were performed with the patients lying supine with their feet placed against a foot plate and their forefeet held together with a rubber band to standardize the position. A coronal isotropic 3D T1-weighted sequence of both ankle and calfs (3D T1w isotrop) was used to measure the Achilles tendon length including the soleus and gastrocnemius tendon components as well as the cross-sectional area (CSA). Lastly, an axial six-point DIXON sequence was applied from the knee to the ankle joint of both calfs (mDixon quant) to measure the free fat fraction of the soleus, medial gastrocnemius, lateral gastrocnemius, and flexor hallucis longus muscles. Horos v3.3.6., an open-source medical image viewer, was used for the MRI tendon length and muscle CSA measurements, and Fiji/ImageJ2 (Version 2.3.0) was used for quantifying the Dixon free fat fraction within the same segmentation used for muscle CSA measurements. For the tendon length measurements, three landmark points in 3D were defined in each lower leg as outlined previously [9, 21]. Landmark (1): The most proximal insertion of the Achilles tendon on the calcaneus. Landmark (2): The most distal part of the myotendinous junction (MTJ) between the soleus and Achilles tendon. Landmark (3): The most distal part of MTJ between the medial gastrocnemius and the Achilles tendon. The CSA of the soleus, medial, and lateral GMs was measured manually at one-third of the length of the triceps surae muscle proximal to landmark 3, while CSA of the flexor hallucis longus muscle was measured 4 cm proximal to landmark 1 [21]. Both the injured leg and the uninjured leg were analyzed. The assessor was blinded to injured/uninjured side.

### 2.5. Isokinetic Strength

The injured leg and the uninjured leg were tested starting with the uninjured leg at both timepoints. Concentric strength followed by eccentric strength in plantar flexion was determined isokinetically (Biodex Medical Systems, Shirley, New York) at 30°s^−1^ throughout the full ankle joint range (from 10° of dorsiflexion to 30° of plantar flexion) with the knee in flexion (40°) and then in extension (0°). Ten submaximal efforts were performed followed by 2 × 5 consecutive maximal plantar flexion contractions. Verbal encouragement was provided for each repetition.

### 2.6. Heel-Rise Test

Heel-rise muscle function was evaluated using a standardized standing heel-rise test [22]. Before the test, the patient completed 5 min of warm-up on an exercise bike followed by 10 bilateral heel-rises. Participants were standing on a bench with their heel over the edge and tests were conducted on one leg at a time starting with the uninjured leg. A linear encoder connected to a computer running the MuscleLab® software (Ergotest Innovation A/S, Norway) was attached with a string to the proximal border of the calcaneus to record height and repetitions. The test was performed with a cadence of 30 rises per minute, and a metronome was used to keep the pace. The assessor was not allowed to cheer the participant during the test. The test ended when the participants stopped, were unable to maintain the frequency, or unable to complete the heel-rise without using compensatory techniques (such as bending their knees or leaning too much against the wall). Maximum repetitions and heel-rise height (cm) were obtained and total work in Joule was calculated. The first and last attempts were removed in the heel-rise height measurements. The test has been shown to be valid and reliable [22].

### 2.7. Achilles Tendon Rupture Score (ATRS)

The participants completed the ATRS, a patient-reported outcome measure that was developed with the involvement of Achilles tendon rupture. ATRS has been reported to have content and construct validity for patients with a total Achilles tendon rupture [23]. The ATRS questionnaire consists of 10 questions evaluating physical activity and symptoms. The maximum score of 100 indicates full physical activity and absence of symptoms.

### 2.8. Training Program

The isokinetic eccentric training program was performed for 12 weeks in a dynamometer (Biodex Medical Systems, Shirley, New York). The program included three 4-week mesocycles and consisted of two weekly training sessions (24 training sessions) with minimum 48 h restitution between consecutive sessions. Weeks 1–4; training volume was gradually increased and consisted of 3 × 10 repetitions with the knee in flexion and 3 × 10 repetitions with the knee in extension in the first 4 weeks. Week 5–8; 4 × 10 repetitions with the knee in flexion and 4 × 10 repetitions with the knee in extension. Weeks 9–12; 5 × 10 repetitions with the knee in flexion and 5 × 10 repetitions with the knee in extension. Only the injured leg was trained while the healthy served as a control. A 10-min warm-up on a cycle ergometer was performed followed by 10 submaximal eccentric contractions at 30 s^−1^ throughout the full ankle joint range in the isokinetic dynamometer. The participant was seated with the hip flexed at 70°. Training was performed with the knee in flexion (40°) first and thereafter with the knee in extension (0°). During training, participants were instructed to resist the dorsiflexion motion generated by the dynamometer using maximal effort contractions with the same range and speed as specified above. Verbal encouragement was provided during each training session. Participants only performed eccentric contractions during the training program. There was a 1 min interval between sets.

### 2.9. Statistics

Based on previous data [16], a sample size of *n* = 15 was required to detect a 13% increase in fascicle length. All data were examined using two-way ANOVA (side × time) with repeated measures except the ATRS score and tendon length measurements, which were analyzed with a paired Students *t*-test. An alpha-level of 0.05 was considered significant. All statistical analyses were completed in GraphPad Prism 9.4.1. Data are reported as 95% CI and as mean ± SEM in the figures unless otherwise stated.

## 3. Results

Four of the 14 participants had their Achilles tendon rupture treated with surgery and 10 without surgery. All participants completed 12 weeks of eccentric training. Five completed all twenty-four training sessions, four participants missed one session, and five missed two sessions. Total compliance was 95%.

### 3.1. Muscle Dimensions

**Figure 1:**
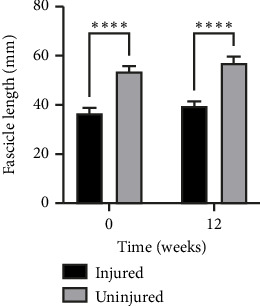
Medial gastrocnemius muscle fascicle length on the injured and uninjured sides. A significant difference (*p* < 0.0001) between injured and uninjured sides was found at both timepoints, and there was a main effect of time (*p*=0.0179). Mean±SEM. ^∗∗∗∗^A significant difference (*p* < 0.0001).

For gastrocnemius fascicle length, there was no interaction, but a significant main effect of side (*p* < 0.0001) and time (*p*=0.0179), with a shorter fascicle length on the injured side and increased fascicle length at 12 weeks (Figure 1). However, post-hoc testing revealed no significant increase with time on the injured (*p*=0.15) or uninjured (*p*=0.11) side separately. For GM thickness, there was no interaction or time effect, but a significant effect of side, *p* < 0.0001, with a smaller muscle on the injured side. For GM pennation angle, there was no interaction, but a significant effect of side (*p*=0.0002) and time (*p*=0.0072) showing a larger pennation angle on the injured side, which decreases over time.

### 3.2. Tendon Length

**Figure 2:**
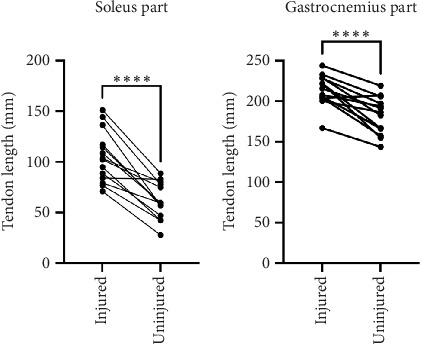
Achilles tendon length on the injured and uninjured sides. (a) The soleus part and (b) the gastrocnemius part were both significantly longer on the injured side than the uninjured side at week 0 (*p* < 0.0001). ^∗∗∗∗^A significant difference (*p* < 0.0001).

The Achilles tendon on the injured leg was significantly longer than the uninjured leg at week 0 for both the soleus part (mean diff. = 44.0 mm, 95% CI [31.7–56.3 mm], *p* < 0.0001) and the gastrocnemius part (mean diff. = 32.0 mm, 95% CI [21.4–42.6 mm], *p* < 0001) of the tendon (Figures 2(a) and 2(b)). The absolute length difference corresponded to 60% elongation for the soleus part and 18% for the gastrocnemius part of the Achilles tendon.

### 3.3. Heel-Rise Test

**Figure 3:**
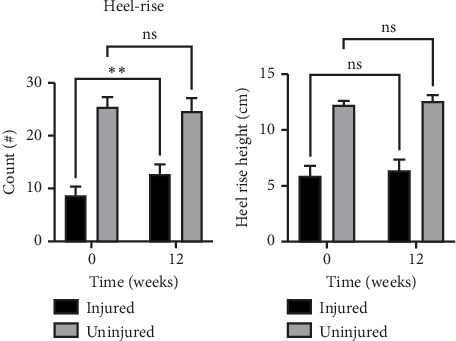
(a) Heel-rise count displayed a significant difference (*p* < 0.0001) between the injured and uninjured sides and an interaction between side and time ^∗∗^(*p*=0.0065). (b) Heel-rise height displayed a significant difference (*p* < 0.0001) between the injured and uninjured sides at both timepoints. Mean±SEM.

At baseline, the injured side displayed a lower heel-rise height (52%, *p* < 0.0001) and a reduced heel-rise count (66%, *p* < 0.0001) than the uninjured side. There was no interaction or time effect, but a significant effect of side (*p* < 0.0001) with a lower mean heel-rise height on the injured side. For heel-rise count, there was a significant interaction effect (*p*=0.0065) and effect of side (*p* < 0.0001), with an increase in mean count on the injured side over time, causing the difference between sides to be significantly lower at 12 weeks (Figures 3(a) and 3(b)).

### 3.4. Plantar Flexion Torque

**Figure 4:**
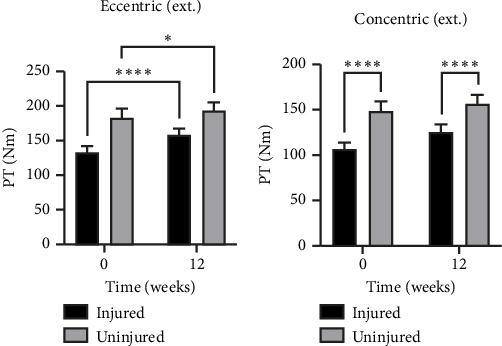
(a) Eccentric plantarflexion peak torque with the knee extended. ^∗∗∗∗^A significant difference (*p* < 0.0001) between injured side at week 0 and 12. ^∗^A significant difference (*p* = 0.0177) between uninjured side at week 0 and 12. (b) Concentric plantarflexion peak torque with the knee extended. ^∗∗∗∗^A significant difference (*p* < 0.0001) between injured and uninjured side was found at both timepoints. Mean ± SEM.

**Figure 5:**
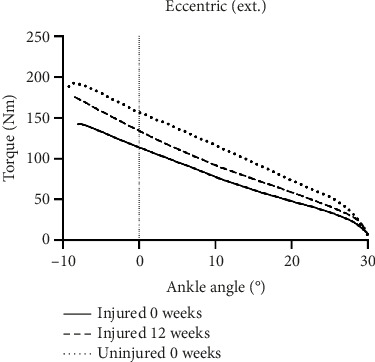
Eccentric plantar flexion torque with the knee extended. Mean values at each ankle angle for the injured side at 0 and 12 weeks and the uninjured side at 0 weeks.

For both eccentric and concentric plantar flexion peak torque performed with the knee extended, there was a significant interaction effect (eccentric: *p*=0.0074 and concentric: *p*=0.0187) and a significant effect of side (eccentric: *p*=0.0002 and concentric: *p* < 0.0001) and time (eccentric *p*=0.0179 and concentric *p*=0.0093): there was a greater increase in peak torque on the injured side over time, which reduced the side-to-side difference at 12 weeks (Figures 4(a) and 4(b)). For eccentric plantar flexion peak torque performed with the knee flexed, there was a significant interaction effect (*p*=0.0480) and a significant effect of side (*p* < 0.0001) but no effect of time. For concentric plantar flexion peak torque performed with the knee flexed, there was no interaction or time effect, but a significant effect of side (*p* < 0.0001) with a lower peak torque on the injured side. Aside from the peak torque, we also assessed the torque versus ankle angle relationship but found no relation between injury or intervention and angle (e.g. the injury did not affect certain angle ranges more than others). To illustrate these data, the torque-angle relation for the eccentric plantar flexion with extended knee is shown in Figure 5.

### 3.5. CSA and Fat Fraction

At baseline, there was a higher fat content in the triceps surae on the affected side compared to the unaffected side (lateral gastrocnemius 44%, *p* < 0.0001, medial gastrocnemius 77%, *p* < 0.0001, and soleus 107%, *p* < 0.0001), For the CSA and fat fraction of the medial gastrocnemius and soleus muscles (Table 1), there was no interaction or time effect, but a significant effect of side, *p* < 0.0001, with a smaller CSA and a higher percentage of fat on the injured side. For the CSA of the lateral gastrocnemius, there was no interaction or side effect but a significant increase over time, *p*=0.0272. Fat fraction of the lateral gastrocnemius displayed no interaction or time effect but a significant effect of side, *p*=0.0316, with a higher percentage of fat at the injured side. There were no significant effects for the flexor hallucis longus muscle.

### 3.6. Achilles Tendon Rupture Score

There was a significant improvement in ATRS (mean ± SD) from week 0 (51 ± 14 points) to week 12 (62 ± 15 points) (mean difference 11.6 points, 95% CI [4–19], *p* < 0.0001).

## 4. Discussion

The aim of the present study was to explore if a muscle that has undergone shortening secondary to elongation of the tendon, and therefore has a reduced overall excursion, could increase its fascicle length and function following eccentric training. In contrast to our hypothesis, while there was an increase in fascicle length after 12 weeks, it was not restricted to the exercised side. However, after 12 weeks of eccentric training, the patient reported outcome measure ATRS, the plantar flexion toque with extended knee, and the heel-rise count all increased significantly. In addition, the data show that persons with a prior Achilles tendon rupture (> 1 year) who were dissatisfied with their function displayed an elongated Achilles tendon, shorter medial GM fascicles, marked atrophy of the soleus and medial GM, a reduced heel-rise height and count, decreased eccentric and concentric plantar flexion muscle strength, and a higher fat content in the triceps surae on the affected side compared to the unaffected side.

Total muscle excursion and thus joint range of motion (ROM) is related to muscle length [24], and it has been shown that the Achilles tendon may elongate while the GM shortens after a rupture, which can affect the heel‐rise height [8, 9, 25, 26]. Fascicle length will increase in healthy individuals in response to eccentric training [16, 27]. However, despite rigorously adhering to the identical training methodology utilized in earlier studies [16], 12 weeks of isolated eccentric exercise had no effect on the fascicular length of the medial gastrocnemius on the injured side in this investigation. The lack of change in fascicle length may be because the participants were unable to generate enough force for a muscle adaptation, which is supported by reduced muscle CSA. Interestingly, the reduced force-generating capacity was most pronounced in the most plantar flexed position, which corresponds to earlier reports [28], and may relate to the shortened fascicle length. Anecdotally, the participants verbally reported that they only started to experience activation of the muscles after ∼8 weeks of training, and therefore it cannot be ruled out that a longer training period would have impacted the fascicle length, which is partly supported by numeric increase in fascicle length of 9%. It is noteworthy that there was an increase in fascicle length on both sides and therefore the change on the affected side cannot solely be ascribed to the training alone. It cannot be excluded that the training increased the overall functional level as supported by the increased ATRS score. For the measurement of the fascicle length, the participant was lying prone with knees straight and feet hanging freely over the edge of the examination table. It cannot be ruled out that if fascicles changed length (& pennation angle) following the intervention, it could influence the passive tension and ankle joint position, which might influence the measurements of the fascicle length.

There is commonly a side-to-side strength deficit after a ruptured Achilles tendon of 10%–35% [2]. In the present study, the participants displayed a rather large side-to-side difference (∼30%) in both eccentric and concentric muscle plantar flexion strength, which is likely due to selection criteria. This rather pronounced strength deficit may also partly explain the low ATRS score (week 0 = 51 ± 14 points, week 12 = 62 ± 15 points), which was much below the average ATRS score of ∼80 reported around 12 months after rupture [2].

It has been shown that in healthy persons, 12 weeks of strength training resulted in increased muscle strength (∼17%) and muscle CSA (type II fiber ∼16%) [29, 30]. The present data show an increase in muscle strength without any increase in muscle CSA, which may indicate that the augmented strength increase was largely a neural phenomenon. Also, we expected that the participants would develop “delayed-onset muscle soreness” (DOMS) since this is a well-known side effect of eccentric strength training [31]. However, to our surprise, the participants did not report any DOMS in the initial weeks of the intervention. However, about 8 weeks into the training, several participants began to experience muscle cramps during training. Therefore, it is possible that training beyond 12 weeks would be associated with increases in CSA.

The training intervention positively affected the muscle endurance measured by the functional heel-rise test as evidenced by the increased number of repetitions completed. The intervention started with a weekly volume of 120 repetitions and ended with a weekly volume of 200 repetitions. Even though both muscle strength and endurance improved, the functional ROM measured as maximal heel-rise height remained unchanged. This lack of improvement may relate to the unchanged fascicle length since muscle fiber length is related to the total muscle excursion and joint ROM [24]. The reduced heel-rise height on the affected side compared to the unaffected side is likely a function of the elongated Achilles tendon coupled with a compensatory shortening of the muscle [8, 9, 25], and therefore this functional deficit is unlikely to be affected by either endurance or strength per se.

The data of the present study demonstrated that the long-term (> 1 year) effects of an Achilles tendon rupture also include a significant increase in the fat fraction of the muscles in the injured side: relative to the uninjured control, the fat fraction was 52% and 43% greater on the injured side in the soleus and medial gastrocnemius, respectively. These data also corroborate earlier findings in a similar population [7, 32, 33]. There is an increased fat infiltration associated with the aging process [34, 35], and with the loss of contractile tension, there is an increase in intramuscular lipid deposition [36, 37]. Therefore, the increased fat deposition in the current study is likely a function of a reduced ability to generate tension secondary to the reduced plantar flexion torque. Numerous studies [7, 33, 38, 39] have shown that there is muscle atrophy associated with an Achilles tendon rupture; however, these data are likely an underestimation of the loss of contractile tissue since some fraction of CSA clearly represents a noncontractile tissue. The intervention did not impact the CSA or fat fraction, which may be because the total training stimulus was too modest. Flexor hallicus longus has been shown to contribute to plantar flexion torque [33, 40, 41]. The flexor hallucis longus has also been shown to have a larger CSA in patients with an Achilles tendon rupture [7] and display a greater relative EMG activity during plantar flexion [42]. In fact, it has been suggested that it may provide some compensatory plantar flexion strength in the early phase of rehabilitation [40]. However, no compensatory hypertrophy of the muscle was observed in this study sample. Moreover, the lack of significant atrophy or fat infiltration indicates that this muscle has been subjected to normal activation. Finally, it is noteworthy that anecdotally the participants verbally reported improvements such as not needing to use a compression sock on the injured leg since it no longer became edematous as it did before the training period or that family and friends of the participants had commented that they had acquired a better gait pattern.

## 5. Conclusion

In conclusion, persons with a prior unilateral Achilles tendon rupture who still experienced functional deficits > 1 year after injury reported improved functional outcome, increased plantar flexion torque, and heel-rise count after 12 weeks of isokinetic eccentric training. However, in contrast to our hypothesis, the training did not alter the fascicle length of the medial gastrocnemius. These data suggest that future larger studies in a similar patient population should consider longer training period.

## Figures and Tables

**Table 1:** Cross-sectional area (CSA), fat fractions (FF), thickness (Thick), pennation angle (Penn), and peak torque (PT) measured at weeks 1 and 12.

	Week 0		Week 12		*p* value		
	Injured	Uninjured	Injured	Uninjured	Time	Side	Time × side
CSA, GL (cm^2^)	7.3 ± 0.8	8.0 ± 0.7	7.8 ± 0.8	8.9 ± 0.7	0.0272	0.0709	0.5292
CSA, GM (cm^2^)	12.0 ± 0.9	16.1 ± 1.2	12.3 ± 1.0	16.3 ± 1.3	0.5014	< 0.0001	0.7793
CSA, SOL (cm^2^)	20.8 ± 1.5	30.0 ± 2.0	21.1 ± 1.7	30.0 ± 2.2	0.7624	< 0.0001	0.6339
CSA, FHL (cm^2^)	3.6 ± 0.3	3.9 ± 0.3	3.7 ± 0.4	4.0 ± 0.3	0.3486	0.2418	0.7767
FF, GL (%)	11.5 ± 1.6	8.0 ± 0.6	11.3 ± 1.8	7.0 ± 0.8	0.5652	0.0316	0.8675
FF, GM (%)	13.8 ± 1.3	7.8 ± 0.8	14.0 ± 1.4	7.5 ± 1.0	0.5353	< 0.0001	0.5435
FF, SOL (%)	14.5 ± 1.5	7.0 ± 0.6	14.5 ± 1.6	7.0 ± 0.7	0.9670	< 0.0001	0.3233
FF, FHL (%)	11.1 ± 0.8	11.5 ± 0.9	11.7 ± 1.5	10.0 ± 0.9	0.3597	0.3621	0.0581
Thick, GM (mm)	15.9 ± 0.7	20.5 ± 0.9	16.5 ± 0.6	20.2 ± 0.9	0.7249	< 0.0001	0.1869
Penn, GM (^o^)	30.2 ± 1.2	23.7 ± 0.9	28.4 ± 1.1	22.5 ± 0.8	0.0072	0.0002	0.5057
PT, flex. C (Nm)	90.5 ± 9.1	138.0 ± 8.0	98.4 ± 8.1	136.6 ± 9.4	0.5976	< 0.0001	0.2461
PT, flex. E (Nm)	115.6 ± 12.4	173.4 ± 12.9	125.6 ± 9.0	166.5 ± 10.6	0.8303	< 0.0001	0.0480

*Note:* Data are presented as mean ± SEM.

Abbreviations: C, concentric; E, eccentric; Flex, knee in flexion; GL, gastrocnemius lateralis muscle; GM, gastrocnemius medialis muscle; FHL, flexor hallucis longus muscle; SOL, soleus muscle.

## Data Availability

The data that support the findings of this study are available from the corresponding author upon reasonable request.
